# LTF promotes central nervous system leukemia progression via neutrophil serine proteases

**DOI:** 10.3389/fphar.2026.1813396

**Published:** 2026-07-01

**Authors:** Yiran Zhang, Huasong Yu, Zhongxiang Jiao, Ming Yang, Wenshan Zhang, Chaoqun Wang, Hexiao Zhang, Wei Wang, Minghe Zhang, Xiaoyue Li, Lin Song, Bo Jiang, Junyuan Qi, Yinghui Li, Yingdai Gao

**Affiliations:** 1 State Key Laboratory of Experimental Hematology, National Clinical Research Center for Blood Diseases, Haihe Laboratory of Cell Ecosystem, Institute of Hematology and Blood Diseases Hospital, Chinese Academy of Medical Sciences and Peking Union Medical College, Tianjin, China; 2 Tianjin Institutes of Health Science, Tianjin, China; 3 Department of Pharmacy, Children’s Hospital of Soochow University, Suzhou, Jiangsu, China

**Keywords:** acute myeloid leukemia, central nervous system leukemia, lactoferrin, neutrophil serine proteases, brensocatib

## Abstract

**Introduction:**

Central nervous system (CNS) infiltration is a severe complication of acute myeloid leukemia (AML), often leading to relapse and poor prognosis. The underlying mechanisms remain poorly understood, limiting the development of effective targeted therapies. Conventional chemotherapy agents capable of crossing the blood-brain barrier (BBB) carry significant toxicity and fail to eliminate leukemia stem cells.

**Methods:**

Using multiple clinical cohorts, we established prognostic prediction model for AML. Using knockdown and overexpression experiments in a mouse leukemia CNS infiltration model, the role of risk factor gene in CNS infiltration was evaluated, along with the attenuating effect of its downstream proteins drug inhibition on leukemia CNS infiltration.

**Results:**

We identify Lactoferrin (LTF) as a risk factor associated with poor AML prognosis using a machine learning–based prognostic model. Through *LTF* knockdown and overexpression in a mouse leukemia model, we demonstrate its pivotal role in CNS invasion. Mechanistically, neutrophil serine proteases (NSPs) act as downstream effectors of LTF, and pharmacological inhibition with Brensocatib effectively blocks AML cell entry into the brain.

**Discussion:**

Our findings establish LTF as a mediator of CNS infiltration in AML and highlight NSP inhibition as a promising therapeutic strategy.

## 1 Introduction

Acute myeloid leukemia (AML) is a malignant clonal disorder originating from hematopoietic stem and progenitor cells and represents the most common acute leukemia in adults, accounting for ∼80% of cases in this population (DiNardo et al., 2023). Beyond bone marrow and peripheral blood, leukemic cells can disseminate via the bloodstream to infiltrate lymph nodes, liver, spleen, and other organs. Critically, they may invade the central nervous system (CNS), causing extramedullary leukemia in the brain and spinal cord. CNS involvement correlates with reduced disease-free survival (DFS) and overall survival (OS) (Burns et al., 2025; Liu et al., 2025) and lower complete remission (CR) rates (Virijevic et al., 2024; Shen et al., 2025), underscoring the need for targeted therapies to prevent recurrence and improve outcomes.

The incidence of CNS involvement differs by leukemia subtype, ranging from 0.6% to 3% in adult AML (Chen et al., 2024) and 5%–11% in acute lymphoblastic leukemia (ALL) (Kopmar and Cassaday, 2023). Not all patients develop CNS disease, reflecting heterogeneity in leukemic cell invasiveness. The molecular drivers of CNS invasion remain poorly defined. Moreover, many chemotherapeutics are ineffective against CNS leukemia due to the blood-brain barrier (BBB) and efflux pumps. Cytarabine and methotrexate, capable of BBB penetration, are standard agents for high-dose systemic and intrathecal chemotherapy (Maya et al., 2025); however, they have severe side effects (Ramanathan et al., 2022; Rupakumar et al., 2024) and do not eradicate leukemia stem cells, resulting in high CNS relapse rates (Zheng et al., 2014; Stelmach and Trumpp, 2023). Current therapies largely target proliferating leukemic cells but fail to address mechanisms of invasion, and remain insufficient for many molecularly and clinically heterogeneous AML subtypes that lack durable or standardized treatment options, thereby highlighting the need to dissect CNS leukemia pathogenesis and develop novel targeted interventions (Bhansali et al., 2023; Fang et al., 2024).

Lactoferrin (LTF) is an iron-binding glycoprotein of the transferrin family, highly expressed in epithelial and myeloid cells and stored in neutrophil secondary granules (Rizzi et al., 2025). Beyond its canonical roles in iron homeostasis and innate immunity, LTF has context-dependent roles in cancer, including both tumor-suppressive functions—such as antiproliferative and pro-apoptotic effects (León-Flores et al., 2025) —and tumor-promoting activities that enhance malignancy and metastasis (Ha et al., 2011; Hoedt et al., 2014; Wang et al., 2024). Notably, unlike in solid tumors, LTF is overexpressed in certain hematologic malignancies, including T-ALL (Tian et al., 2023) and B-ALL (Xiao et al., 2025), and primary CNS diffuse large B-cell lymphoma (DLBCL) (Lemma et al., 2017), which frequently infiltrate the CNS. These observations suggest LTF may facilitate CNS invasion in leukemia, yet its mechanistic role remains unexplored.

In this study, using a machine learning-based AML prognostic model constructed from a large patient cohort, we identified LTF as an AML risk factor associated with poor prognosis and inferred that it may be involved in CNS AML. Functional experiments in AML mouse models reveal that LTF, through downstream neutrophil serine proteases (NSPs) including CTSG and PRTN3, promotes CNS infiltration. Pharmacological inhibition of NSPs effectively blocks leukemic cell entry into the CNS, indicating a potential therapeutic avenue. By integrating bioinformatics, molecular biology, and pharmacological approaches, our study elucidates mechanisms driving CNS leukemia and identifies actionable targets for intervention.

## 2 Materials and methods

### 2.1 Cell line

HEK293T cells were cultured in Dulbecco’s Modified Eagle Medium (DMEM) (Gibco, Cat#11995065) supplemented with 10% fetal bovine serum (FBS) (Gibco, Cat#16000044) and 1% penicillin/streptomycin (Solarbio, Cat#P1400-100). Human AML cell lines HL60 and THP1 were cultured in Roswell Park Memorial Institute 1,640 Medium (RPMI 1640) (Gibco, Cat#11875093) supplemented with 10% FBS and 1% penicillin/streptomycin. All cells were incubated at 37 °C in a humidified atmosphere with 5% CO_2_.

### 2.2 Primary AML patient sample

Bone marrow aspirates were obtained from patient with AML at the Institute of Hematology and Blood Diseases Hospital (Tianjin, China). Bone marrow mononuclear cells (MNCs) were isolated by density gradient centrifugation using Lymphocyte Separation Medium (Tbd Science, Cat#LTS1077), according to the manufacturer’s instructions. Isolated MNCs were cryopreserved in serum-free freezing medium BAMBANKER (FUJIFILM, Cat#302-14686) and stored in liquid nitrogen until use.

For *in vitro* culture, primary human cells were maintained in Iscove’s Modified Dulbecco’s Medium (IMDM) (Gibco, Cat#12440053) supplemented with 10% fetal bovine serum (FBS) (Gibco, Cat#16000044), 100 ng/mL recombinant human stem cell factor (rhSCF) (PeproTech, Cat#300-07), 100 ng/mL recombinant human Flt3 ligand (rhFlt3L) (PeproTech, Cat#300-19), 100 ng/mL recombinant human thrombopoietin (rhTPO) (PeproTech, Cat#AF-300-18-10), and 1% penicillin/streptomycin (Solarbio, Cat#P1400-100). Cells were cultured at 37 °C in a humidified incubator with 5% CO_2_.

Experiment involving human samples was conducted in accordance with the Declaration of Helsinki and was approved by the Ethics Review Board of the Institute of Hematology and Blood Diseases Hospital, Chinese Academy of Medical Sciences (Approval No. KT2020024-EC-2). Detailed clinical information for all patient samples included in this study is provided in the Supplementary Table S4.

### 2.3 Animals

C57BL/6 J mice were obtained from Beijing HFK Bioscience Co., Ltd. Eight-week-old female mice were randomly assigned to experimental groups and maintained in a specific pathogen-free (SPF) facility with *ad libitum* access to food and water. All animal procedures were approved by the Animal Care and Use Committee of the State Key Laboratory of Experimental Hematology, Institute of Hematology and Blood Diseases Hospital (Approval No. IHCAMS-DWLL-NSFC2024010-1).

### 2.4 Plasmid construction

MSCV-MLL-AF9-IRES-GFP, pMD2. G, psPAX2, pKat, and pVSV-G plasmids were obtained from Yingchi Zhang’s laboratory (Feng et al., 2021). Lentiviral shRNA knockdown and overexpression vectors were obtained from YouBio (China). The pLVX-U6-mCherry-Puro vector encoded shRNAs targeting mouse *LTF*, with sequences listed in Supplementary Table S3. For overexpression, full length *LTF* cDNA was cloned into the pCDH-SFFV-mCherry-Puro vector, all constructs were verified by Sanger sequencing.

### 2.5 Lentivirus and retrovirus production and infection

Lentiviral and retroviral particles were produced by transient transfection of HEK293T cells using Hieff Trans® Liposomal 2000 (Yeason, Cat#40802ES03), according to the manufacturer’s protocol.

For lentiviral packaging, transfer plasmids were co-transfected with pMD2.G and psPAX2 at a ratio of 7:3:5, while retroviral packaging used pKat and pVSV-G at a ratio of 8:4:3. Viral supernatants were collected at 48 h and 72 h post-transfection, filtered through 0.45 μm syringe filters (Pall, Cat#4614), and concentrated overnight at 4 °C with 4 × PEG-6000 (Solarbio, Cat#P8250). Viral particles were pelleted by centrifugation at 1,600 × *g* for 60 min at 4 °C and resuspended in 300 μL of medium.

Target suspension cells were infected with viral supernatants in the presence of 6 μg/mL polybrene via two rounds of spinoculation (1800 rpm, 90 min) at 33 °C. For lentiviral transduction, stable lentivirus-infected clones were selected using 6 μg/mL puromycin, beginning 48 h post-infection and maintained under selection using 3 μg/mL puromycin.

### 2.6 Generation and analysis of the murine MLL-AF9 leukemia model

For *LTF* overexpression in MA9_WT cells, bone marrow (BM) was harvested from the femurs, tibias and iliums of 6–8-week-old female C57BL/6J mice. c-kit^+^ cells were enriched using CD117 microbeads (Miltenyi Biotec, Cat#130-091-224) according to the manufacturer’s instructions and preconditioned for 12 h in complete medium (IMDM supplemented with 15% FBS and 1% penicillin/streptomycin) supplemented with 10 ng/mL rmIL-3 (PeproTech, Cat#213-13), 10 ng/mL rmIL-6 (PeproTech, Cat#216-16), and 50 ng/mL rmSCF (PeproTech, Cat#250-03). Cells were then infected twice with MSCV-MLL-AF9-IRES-GFP retrovirus in the presence of 6 μg/mL polybrene (Beyotime Biotechnology, Cat#C0351), maintaining the cytokine combination during infection. Each infection was performed for 6–8 h, with centrifugation at 1800 rpm for 90 min.

For primary transplantation (P0), 1 × 10^6^ infected cells were injected into lethally irradiated (8 Gy, split as two 4 Gy fractions, 6 h apart) C57BL/6J recipients (*n* = 6 per group). For serial transplantation, 2 × 10^5^ BM-derived leukemia cells from P0 mice were transplanted into sub-lethally irradiated (4 Gy) recipients (*n* = 6 per group). P3-generation BM cells were used for LTF overexpression or empty vector (EV) control in MA9_WT cells via the lentiviral protocol described above.

For *LTF* knockdown, MA9_*Irf7* ^*−/−*^ cells (kindly provided by Guoguang Zheng’s lab (Wang H. et al., 2022)) were transduced with shLtf or scrambled control (shNC) plasmids following the same lentiviral infection procedure. The target sequences of shLtf are listed in Supplementary Table S3.

To establish CNS AML models, 5 × 10^5^ shRNA-infected MA9_*Irf7* ^*−/−*^ cells or MA9_WT cells overexpressing LTF were injected via the tail vein without irradiation, as previously described for CNS infiltration models (*n* = 6 per group). Peripheral blood (PB) was collected biweekly from day 14 post-transplantation to monitor leukemia burden by flow cytometry based on GFP^+^ leukemic cells. When PB leukemia reached 10%–15%, mice were sacrificed, and brains were harvested, fixed in formalin, embedded in paraffin, and sectioned for hematoxylin-eosin (HE) staining. CNS infiltration was quantified in brain sections under microscopy using number of fields.

### 2.7 Brensocatib treatment

To inhibit CTSG and PRTN3 activity, mice transplanted with 5 × 10^5^ MA9_*Irf7* ^*−/−*^ cells were randomly assigned to vehicle or treatment groups (*n* = 6 per group). Brensocatib (Selleck, Cat#E1188) was administered by oral gavage at 30 mg/kg/day, five times per week, starting on day 14 post-transplantation. Control mice received an equal volume of vehicle (5% DMSO, 40% PEG-300, 55% PBS).

Treatment was continued until a predefined experimental endpoint, defined as PB leukemia burden reaching 10%–15%, as assessed by flow cytometry. At the endpoint, mice were sacrificed and brains were then harvested and analyzed via HE staining. CNS infiltration was evaluated by quantifying leukemic cell infiltration in brain sections under microscopy.

### 2.8 Flow cytometry analysis

Single-cell suspensions were prepared from BM, spleen, and PB. PB samples were treated with RBC lysis buffer (Solarbio, Cat#R1010) according to the manufacturer’s instructions before analysis. Leukemia burden was quantified by measuring the percentage of GFP^+^ cells using flow cytometry (BD LSRFortessa, BD Biosciences). At least 5,000 viable single-cell events were collected per sample. For viability assessment, DAPI was added immediately before acquisition to exclude dead cells.

Data were analyzed with FlowJo Software (v10). Cells were first gated based on forward and side scatter to exclude debris, followed by doublet and death cell exclusion, then identification of GFP^+^ leukemic cells. The same gating strategy was applied to all samples.

### 2.9 Cell proliferation and colony-forming unit (CFU) assays

For proliferation assays, 5 × 10^4^ primary MLL-AF9–derived leukemia cells were seeded in 1 mL complete medium per well of a 24-well plate and cultured for 72 h. Cells were gently resuspended before sampling. Every 24 h, 10 μL of cell suspension was collected and mixed with an equal volume of trypan blue (Solarbio, Cat#C0040), viable cells were counted using an automated cell counter. Cell growth curves were generated based on viable cell counts.

For colony-forming unit (CFU) assays, 300 primary MLL-AF9 leukemia cells were plated in MethoCult M3434 methylcellulose medium (StemCell Technologies, Cat#03434) according to the manufacturer’s instructions. Colonies consisting of ≥50 cells were counted after 7–12 days as positive colonies. Images were acquired using the Operetta CLS™ imaging system (PerkinElmer). Colony numbers were quantified using manual counting.

### 2.10 Transwell invasion assay

Transwell invasion assays were performed using 24-well Transwell inserts with 8 μm pore size (BeyoGold™, Beyotime, Cat#FTW067-12Ins). Inserts were coated with 60 μL of Matrix-Gel™ diluted 1:10 in serum-free medium (Beyotime, Cat#C0372) and gelled at 37 °C for 2 h to allow gel formation. Leukemia cells were resuspended in serum-free medium at 4 × 10^5^ cells per well and seeded into the upper chamber. The lower chamber was filled with 600 μL medium containing chemoattractant, consisting of 15% FBS supplemented with cytokines (10 ng/mL rmIL-3, 10 ng/mL rmIL-6, and 50 ng/mL rmSCF) for MA9 cells, 10% FBS for human leukemia cell lines, or 10% FBS supplemented with cytokines (100 ng/mL rhSCF, 100 ng/mL rhFlt3L, 100 ng/mL rhTPO) for primary AML patient cells. After 24 h incubation at 37 °C, invading cells were collected from the lower chamber, quantified by directly counting the cells in the lower chamber using an automated cell counter and microscopically photographed.

For pharmacological inhibition experiments, leukemia cells were treated with the cathepsin G inhibitor I (Selleck, Cat#E4453, 2 μM) added to the upper chamber at the time of seeding. An equal volume of vehicle was used as control. Cells were incubated for 24 h under the same conditions as described above. After incubation, invading cells in the lower chamber were collected and quantified as described above.

### 2.11 RNA extraction and real-time quantitative PCR (RT-qPCR) analysis

Total RNA was extracted using TRIzol reagent (Invitrogen, Cat#15596018CN) following the manufacturer’s instructions. RNA concentration and purity were assessed using a NanoDrop 2000 spectrophotometer (NanoDrop Technologies). Samples with an A260/A280 ratio between 1.8 and 2.0 were used for subsequent analysis. For cDNA synthesis, 1–2 μg of RNA was subsequently reverse-transcribed using ABScript III RT Master Mix with gDNA Remover (ABclonal, Cat#RK20429) and anchored oligo (dT)20 primers according to the manufacturer’s protocol.

Quantitative real-time PCR (RT-qPCR) was performed in 10 μL reactions in triplicate using 2× Universal SYBR Green Fast qPCR Mix (ABclonal, Cat#RK21203) on a QuantStudio five system (Thermo Fisher Scientific). Each reaction contained 2 μL cDNA template and 0.2 μM primers. Cycling conditions were: 95 °C for 2 min, followed by 40 cycles of 95 °C for 5 s and 60 °C for 34 s. A melting curve analysis was performed to confirm the specificity of amplification. Gene expression levels were normalized to *GAPDH*, and relative expression was calculated using the 2^−ΔΔCT^ method. Primer sequences are listed in Supplementary Table S2.

### 2.12 Protein preparation and immunoblotting

Cells were lysed in RIPA buffer (Solarbio, Cat#R0020) supplemented with protease inhibitors (Yeason, Cat#20124ES03) on ice for 30 min, vortexing every 10 min. Lysates were centrifuged at 12,000 × *g* for 10 min at 4 °C, and the supernatant was collected as total protein. Protein concentration was determined using a BCA Protein Assay Kit (Thermo Scientific, Cat#23227). Equal amounts of protein were mixed with 5× loading buffer (ABclonal, Cat#RM00001) and denatured at 95 °C for 10 min.

20–30 μg proteins were separated on 4%–20% SurePAGE gels (GenScript, Cat#M00657) and transferred to PVDF membranes (Millipore, Cat#IPFL00010). Membranes were blocked for 1 h at room temperature (RT) with TBST containing 5% BSA (Solarbio, Cat#A8020). Primary antibodies were diluted in blocking buffer and incubated overnight at 4 °C with gentle agitation. Primary antibodies are listed in Supplementary Table S1. After three 5-min washes with TBST, membranes were incubated with HRP-conjugated secondary antibodies for 1 h at RT, followed by three additional washes. Protein signals were detected using ultra-sensitive ECL substrate (CotyBioTech, Cat#KE0126) and imaged on a ChemiDoc system (Bio-Rad).

### 2.13 RNA-seq data processing

Four independent public datasets were collected from The Cancer Genome Atlas (TCGA, https://portal.gdc.cancer.gov/) and the Gene Expression Omnibus (GEO, https://www.ncbi.nlm.nih.gov/geo/), including TCGA-LAML, GSE37642, GSE12417, and GSE106291 (Metzeler et al., 2008; Cancer Genome Atlas Research et al., 2013; Herold et al., 2014; Herold et al., 2018). Only datasets with available gene expression profiles and complete overall survival (OS) information were included in this study. These four cohorts were ultimately enrolled for the development and validation of the prognostic model. For TCGA-LAML, RNA-seq expression data were downloaded and transformed into TPM values, followed by log2 normalization. For the GEO datasets, the normalized expression matrices were downloaded directly from the GEO portal. To minimize inter-cohort technical variability, expression data from all cohorts were further processed, normalized, and adjusted for batch effects before downstream analyses, following a previously reported strategy (Liu et al., 2022). Because the primary purpose of this study was to identify prognostic genes and construct a gene expression-based prognostic signature, the gene expression matrix and OS data were incorporated into the machine learning model. The genes used for model construction were extracted based on GO mapping using the clusterProfiler v4.10.0 and GO. db v3.18.0 packages. In addition, we uploaded a script that enables retrieval of genes within a corresponding gene set according to a given GO ID (https://github.com/HuasongYu/GO_Gene_Search).

Ten machine learning algorithms (random survival forest, least absolute shrinkage and selection operator, gradient boosting machine, survival support vector machine, supervised principal components, ridge regression, partial least squares regression for Cox, CoxBoost, stepwise Cox regression, and elastic net) were integrated to generate 101 model combinations for prognostic signature construction. Leave-one-out cross-validation (LOOCV) is a resampling strategy in which one sample is iteratively held out as the test set, while the remaining samples are used for model training. By repeating this procedure for each sample, LOOCV enables efficient use of limited data and provides an estimate of model generalizability. In this study, the four cohorts (TCGA-LAML, GSE37642, GSE12417, and GSE106291) were randomly divided into training and testing subsets at a ratio of 3:1, and model performance was subsequently evaluated using the LOOCV strategy. Using prognosis-related gene expression profiles and OS data, candidate models were constructed in the training sets and subsequently evaluated in the testing sets. For each model configuration, predictive performance was assessed using Harrell’s concordance index (C-index) across the validation cohorts. The model with the best overall performance was selected as the final prognostic signature.

### 2.14 Statistical analysis

Data analysis was performed using GraphPad Prism 9 (GraphPad Software, Inc.), and results were expressed as means ± SD. Statistical significance was assessed using two-tailed, unpaired Student’s *t*-tests. Kaplan-Meier survival curves were compared using the log-rank test. The *P*-value of less than 0.05 was considered statistically significant. In the figures, significance is indicated as *P < 0.05, **P < 0.01, ***P < 0.001, and ****P < 0.0001.

### 2.15 Data availability statement

All data supporting the conclusions of this study are included within the article. Relevant information regarding the prognostic models has been made publicly available on GitHub (https://github.com/HuasongYu/MachineLearn). Additional information or specific data requests can be directed to the corresponding authors.

## 3 Results

### 3.1 *LTF* is a high-weight gene in the AML machine learning based prognostic model

CNS infiltration is a major complication of AML, linked to poor prognosis and higher relapse rates. While multiple prognostic models exist for AML, robust predictors for CNS invasiveness remain lacking. Identifying genes that drive CNS invasion is critical for understanding CNS leukemia pathogenesis and developing targeted therapies. Previous studies in CNS lymphoma suggest that enrichment of genes in the cytokine-related pathway GO:0001817 may underlie CNS invasiveness in hematologic malignancies (Tun et al., 2008).

**FIGURE 1:**
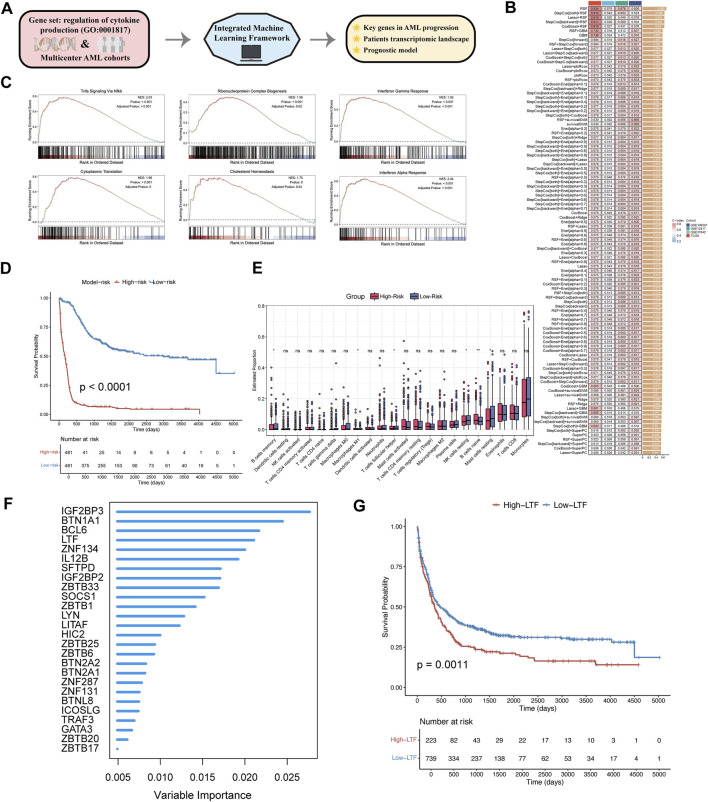
A multicenter cohort-based machine learning approach for discovering key genes of AML progression. **(A)** Flowchart of the screening and analytical procedure. **(B)** Performance comparison of 101 prognostic models generated from 10 machine learning algorithms across all cohorts. Model performance was assessed using Harrell’s concordance index (C-index), where a higher C-index indicates better agreement between predicted risk and observed survival outcome. **(C)** Top six hallmark pathways significantly enriched in the high-risk group, identified by pathway enrichment analysis. **(D)** Kaplan-Meier curves of overall survival (OS) in the Meta-AML cohort stratified by the machine learning-derived risk score. Patients were divided into high-risk and low-risk groups according to the prognostic model, and survival differences between groups were compared. **(E)** Comparison of tumor microenvironment immune cell proportions between the high-risk and low-risk AML groups. The figure shows the relative abundance of infiltrating immune cell populations in the two groups. **(F)** Variable importance of prognostic genes in the RSF model, indicating the relative contribution of each gene to model construction and risk prediction. **(G)** KM curves of OS in the Meta-AML cohort stratified according to LTF expression.

To explore potential genes associated with CNS invasion in AML from the perspective of upstream regulatory pathways, we constructed a prognostic model integrating four independent AML cohorts (TCGA-LAML, GSE37642, GSE12417, and GSE106291) using 101 machine learning algorithms (Figure 1A). This approach leverages a larger sample size and broader algorithmic diversity compared with conventional cancer prognostic models, enabling more objective and comprehensive survival predictions (Liu et al., 2022; Wang et al., 2022b).

Using a LOOCV strategy, we trained and evaluated 101 prognostic models derived from 10 machine learning methods across all validation cohorts (Liu et al., 2022; Wang et al., 2022b). Model performance was quantified using Harrell’s concordance index (C-index), which reflects the consistency between predicted risk and observed survival outcomes. A C-index of 0.5 indicates random prediction, whereas a value of 1.0 indicates perfect predictive accuracy (Harrell et al., 1982); thus, higher values represent better prognostic performance. Among all candidate models, the random survival forest model achieved the highest average C-index (0.699) across the validation cohorts and showed the best performance in each cohort (Figure 1B). Random survival forest is a tree-based ensemble survival model that integrates results from multiple survival trees built on bootstrap samples and random subsets of variables, enabling robust prediction in the presence of censored survival data and complex gene-expression patterns (Gerussi et al., 2022). Accordingly, the random survival forest model was selected as the final prognostic model. Based on the risk scores generated by this model, patients were subsequently classified into high-risk and low-risk groups. High-risk patients exhibited activation of multiple oncogenic pathways (Figure 1C), reduced overall survival (Figure 1D), and a lower abundance of resting NK cells (Figure 1E), indicating an aggressive tumor microenvironment.

Among the genes contributing to the model, *IGF2BP3*, *BTN1A1*, *BCL6*, *LTF*, and *ZNF134* displayed relatively high variable importance, suggesting that they contributed substantially to risk prediction (Figure 1F). Among these genes, *LTF* has rarely been reported as a prognostic marker in AML. In our large AML cohort, high *LTF* expression correlated with poorer clinical outcomes (Figure 1G). Notably, because the model was established on the basis of CNS leukemia-related enriched genes, its predictive capacity may extend beyond conventional survival stratification and provide additional clues for identifying AML patients at higher risk of CNS leukemia involvement. Together, our model demonstrated robust prognostic performance in AML and effectively highlighted key genes linked to CNS leukemia-associated biological characteristics during disease progression. Among these candidate genes, LTF was inferred to be a particularly important prognostic factor and a potential indicator of CNS leukemia risk. In this study, LTF warrants further experimental validation to elucidate its functional role in AML progression and CNS involvement.

### 3.2 *LTF* knockdown attenuates AML cell invasiveness *in vitro*

**FIGURE 2:**
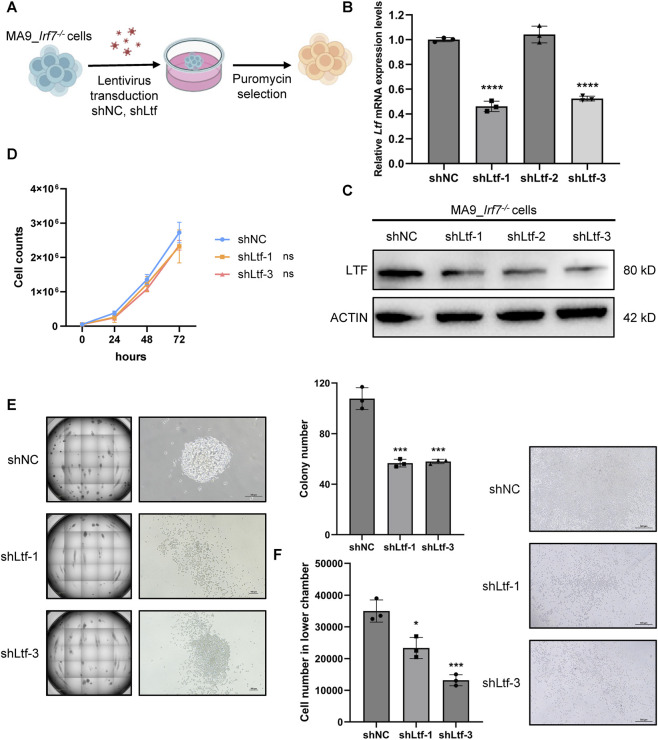
LTF knockdown reduces AML cell invasiveness *in vitro* **(A)** Schematic illustration of lentiviral-mediated LTF knockdown in MA9_*Irf7* ^*−/−*^ cells followed by puromycin selection. **(B)** Relative Ltf mRNA expression levels in MA9_*Irf7* ^*−/−*^ cells transduced with control shRNA (shNC) or Ltf-targeting shRNAs (shLtf-1, shLtf-2, shLtf-3), determined by RT–qPCR after puromycin selection. Expression levels were normalized to Gapdh and are presented relative to the shNC group. **(C)** Immunoblot of LTF protein expression levels in shNC and shLtf cells (ACTIN as loading control). **(D)** Cell proliferation curves of shNC, shLtf-1, and shLtf-3 cells over 72 h, assessed by viable cell counting (n = 3). **(E)** Colony-forming unit (CFU) assay of shNC, shLtf-1, and shLtf-3 MA9_*Irf7* ^*−/−*^ cells: representative microscopy images (left) and colony counts (right) were shown (n = 3). Colonies consisting of ≥50 cells were counted. **(F)** Transwell invasion assay of shNC, shLtf-1, and shLtf-3 MA9_*Irf7* ^*−/−*^ cells. Invading cells were collected from the lower chamber and quantified by cell counting (left) and representative images (right) were shown (n = 3). Data are presented as means ± SD. Statistical significance was determined using two-tailed Student’s t-test: *P < 0.05, ***P < 0.001, ****P < 0.0001; ns, not significant.

Although high *LTF* expression correlated with poor prognosis, its role in CNS infiltration remained unclear. To examine this, we used the MA9_*Irf7* ^*−/−*^ mouse CNS leukemia model, which has been previously reported to reliably recapitulate CNS infiltration in AML, thus serves as a suitable model for investigating CNS leukemia infiltration (Wang H. et al., 2022), and knocked down *LTF* with shRNA (Figure 2A). Knockdown efficiency of three shLtf constructs was assessed by qRT-PCR and Western blot; shLtf-1 and shLtf-3, which showed the most significant reductions, were selected for further experiments (Figures 2B,C).

Trypan blue assays revealed only a slight, non-significant decrease in proliferation of shLtf cells compared to shNC controls over 72 h (Figure 2D), indicating that *LTF* has minimal impact on cell proliferation. CFU assays demonstrated a partial reduction in clonogenic potential following LTF knockdown, with fewer leukemic colonies forming in shLtf cells (Figure 2E). Importantly, Transwell invasion assays showed that *LTF* knockdown significantly decreased the number of leukemia cells migrating through matrixgel-coated (Li et al., 2020) chambers compared to controls (Figure 2F), highlighting its critical role in AML invasiveness.

These results suggest that while *LTF* minimally affects proliferation, it is essential for maintaining clonogenicity and driving the invasive capacity of AML cells *in vitro*.

### 3.3 Silencing *LTF* reduces AML infiltration *in vivo*

**FIGURE 3:**
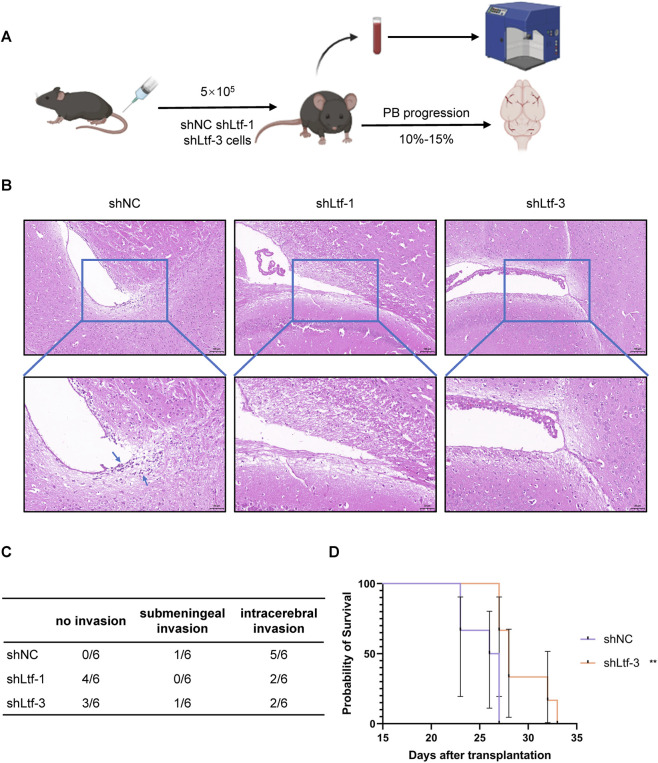
LTF silencing limits AML CNS infiltration *in vivo* **(A)** Schematic illustration of CNS AML model in LTF knockdown mice. MA9_*Irf7* ^*−/−*^ leukemia cells transduced with shNC, shLtf-1, or shLtf-3 were transplanted into recipient mice (5 × 10^5^ cells per mouse via tail vein injection, n = 6). Peripheral blood (PB) leukemia burden was monitored, and mice were sacrificed when PB leukemia reached 10%–15% for downstream analysis. **(B)** Representative hematoxylin and eosin (HE) staining of brain tissue from shNC, shLtf-1, and shLtf-3 mice (n = 6). Low-magnification images are shown to provide anatomical context, with boxed regions enlarged in the corresponding panels. Infiltrating AML cells in the brain tissue are indicated by blue arrows. **(C)** Quantification of leukemic infiltration in the CNS of mice (n = 6). CNS involvement was categorized as no invasion, submeningeal invasion, or intracerebral invasion based on histopathological evaluation of brain sections. Data are presented as the number of mice in each category. **(D)** Kaplan-Meier survival curves comparing shNC and shLtf mice (n = 6). Survival curves are presented with 95% confidence intervals. Statistical significance was determined using the log-rank test: **P < 0.01.

To validate the *in vitro* findings, we assessed CNS infiltration *in vivo* using the MA9_*Irf7* ^*−/−*^ model. Mice were transplanted with shNC or shLtf cells, and brain tissue was collected when peripheral blood leukemia burden reached 10%–15% (Figure 3A). *LTF* knockdown markedly reduced CNS infiltration: all mice in the shNC group showed brain involvement (1/6 submeningeal, 5/6 parenchymal), whereas 4/6 and 3/6 mice in the shLtf groups exhibited no brain infiltration (Figures 3B,C). These results indicate that *LTF* silencing significantly limits leukemia cell CNS invasion, highlighting its potential as a therapeutic target.

Furthermore, survival analysis showed that mice transplanted with shLtf-3 cells had significantly prolonged survival compared to shNC controls (Figure 3D). Collectively, these *in vitro* and *in vivo* results demonstrate that *LTF* knockdown modestly affects proliferation but substantially reduces CNS infiltration, diminishes malignant clonogenicity, and improves survival outcomes.

### 3.4 *LTF* overexpression enhances AML cell invasion

**FIGURE 4:**
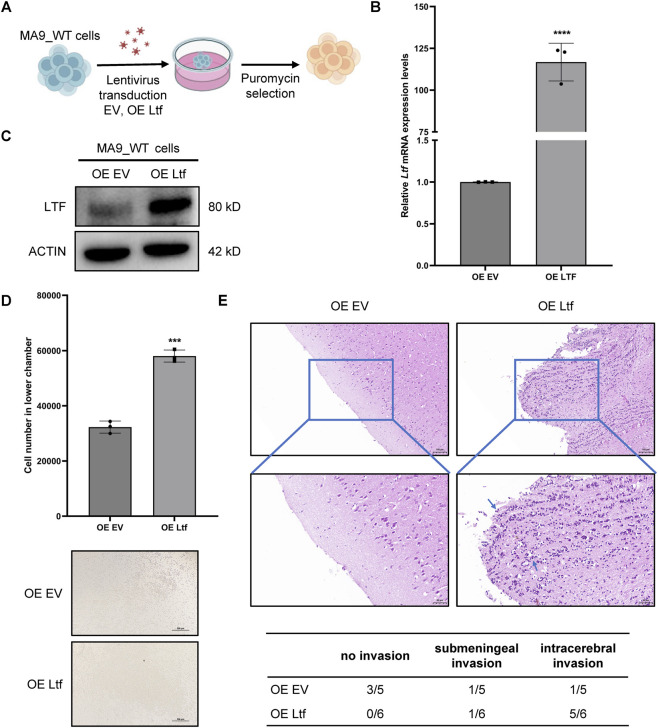
LTF overexpression enhances AML cell invasion and CNS infiltration. **(A)** Schematic of lentiviral-mediated LTF overexpression in MA9_WT cells followed by puromycin selection. **(B)** Relative Ltf mRNA expression levels in MA9_WT cells transduced with empty vector (EV) or LTF overexpression construct (OE Ltf), determined by RT–qPCR after puromycin selection. Expression levels were normalized to Gapdh and are presented relative to the EV group. **(C)** Immunoblot of LTF protein expression levels in EV and OE LTF cells (ACTIN as loading control). **(D)** Transwell invasion assay of EV and OE Ltf MA9_WT cells. Cells were seeded in the upper chamber, and invading cells were collected from the lower chamber after 24 h and quantified by cell counting (upper). Representative images of invaded cells were shown (lower) (n = 3). **(E)** Upper: Representative HE staining of brain tissue from mice transplanted with EV (n = 5) or OE Ltf (n = 6) MA9_WT cells. Low-magnification images are shown to provide anatomical context, with boxed regions enlarged in the corresponding panels. Infiltrating AML cells in the brain tissue are indicated by blue arrows. Lower: Quantification of CNS leukemic infiltration in mice transplanted with EV or OE Ltf cells. CNS involvement was categorized as no invasion, submeningeal invasion, or intracerebral invasion based on histopathological evaluation. Data are presented as the number of mice in each category. Data are presented as means ± SD. Statistical significance was determined using two-tailed Student’s t-test: ***P < 0.001, ****P < 0.0001.

Our previous results demonstrated that LTF knockdown diminishes AML cell invasiveness. To further validate *LTF*’s role in CNS infiltration, we overexpressed *LTF* in wild-type MLL-AF9 (MA9_WT) leukemia cells (Figure 4A). Efficient overexpression was confirmed at both the mRNA (Figure 4B) and protein levels (Figure 4C).

Using Transwell invasion assays, *LTF*-overexpressing MA9_WT cells displayed markedly increased migration through matrixgel compared with empty vector (EV) controls (Figure 4D), demonstrating enhanced invasive potential.

We then assessed CNS infiltration *in vivo* using a mouse transplantation model. Brain tissues were collected when peripheral blood leukemia burden reached 10%–15%. *LTF* overexpression significantly increased AML cell infiltration into the brain (Figure 4E, upper). While 3/5 mice in the EV group showed no brain infiltration, all *LTF*-overexpressing mice exhibited CNS involvement, including 1/6 submeningeal and 5/6 intracerebral infiltration (Figure 4E, lower).

These findings indicate that *LTF* overexpression promotes AML cell invasiveness and CNS infiltration, identifying *LTF* as a risk factor for CNS leukemia.

### 3.5 *LTF*-driven AML invasion is mediated by neutrophil serine proteases

To elucidate the mechanism underlying LTF-driven CNS invasion, we focused on neutrophil serine proteases (NSPs) Cathepsin G (CTSG) and Proteinase 3 (PRTN3), which are co-expressed with LTF in myeloid granules (Hidalgo and Casanova-Acebes, 2021). NSPs are enriched in AML and implicated in extracellular matrix degradation and tissue infiltration (Xu et al., 2024; Okura et al., 2025), suggesting they may mediate LTF-dependent invasiveness.

**FIGURE 5:**
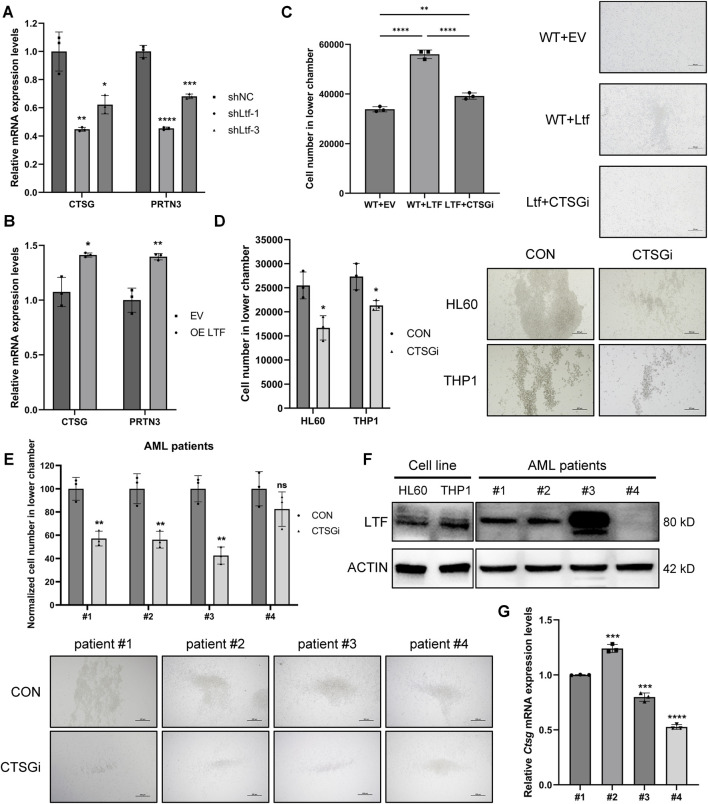
LTF-driven AML invasion is mediated by serine protease activity. **(A)** Relative mRNA expression levels of Ctsg and Prtn3 in MA9_*Irf7* ^*−/−*^ cells transduced with control shRNA (shNC) and shLtf cells determined by RT–qPCR. Expression levels were normalized to Gapdh and are presented relative to the shNC group. **(B)** Relative mRNA expression levels of Ctsg and Prtn3 mRNA in MA9_WT cells transduced with empty vector (EV) and OE LTF cells determined by RT–qPCR. Expression levels were normalized to Gapdh and are presented relative to the EV group. **(C)** Transwell invasion assay of MA9_WT cells transduced with EV or OE LTF, with or without treatment with the cathepsin G inhibitor I (CTSGi). Cells were seeded in the upper chamber, and invading cells were collected from the lower chamber after 24 h and quantified by cell counting (upper, n = 3). Representative images (lower) for EV, OE LTF, and OE LTF + Cathepsin G inhibitor cells are shown. **(D)** Transwell invasion assays of human leukemia cell lines treated with CTSGi. HL60 cells (above) and THP1 cells (below) were treated with 2 μM CTSGi or vehicle control (CON) and seeded in the upper chamber. After 24 h, invading cells in the lower chamber were collected and quantified by cell counting (left, n = 3). Representative images of invaded cells are shown (right). **(E)** Transwell invasion assay of four independent primary AML patient–derived cells treated with 2 μM CTSGi or vehicle control (CON). Cells were seeded in the upper chamber, and invading cells were collected from the lower chamber after 24 h and quantified by cell counting (above, n = 3). Representative images of invaded cells are shown (below). **(F)** Immunoblot of LTF protein expression levels in four independent primary AML patient–derived cells (ACTIN as loading control). **(G)** Relative mRNA expression levels of Ctsg mRNA in four independent primary AML patient–derived cells determined by RT–qPCR. Expression levels were normalized to Gapdh and are presented relative to the AML patient #1. Data are presented as means ± SD. Statistical significance was determined using two-tailed Student’s t-test: *P < 0.05, **P < 0.01, ***P < 0.001, ****P < 0.0001.

RT-qPCR analysis showed that *LTF* knockdown in MA9_*Irf7* ^*−/−*^ cells markedly reduced CTSG and PRTN3 expression, whereas *LTF* overexpression in MA9_WT cells upregulated both NSPs (Figures 5A,B). These results indicate that LTF positively regulates *CTSG* and *PRTN3* expression.

To test whether NSPs mediate LTF-driven invasion, we treated *LTF*-overexpressing MA9_WT cells with 2 μM Cathepsin G inhibitor I (Park et al., 2016). Transwell invasion assays revealed that CTSG inhibition significantly attenuated the enhanced invasiveness conferred by *LTF* overexpression (Figure 5C), confirming that CTSG contributes to LTF-dependent invasion.

To further validate the role of NSP activity in AML cell invasion across different cellular contexts, we treated human leukemia cell lines HL60 and THP1 with 2 μM Cathepsin G inhibitor I. Transwell invasion assays showed that CTSG inhibition significantly reduced the invasive capacity of both HL60 and THP1 cells (Figure 5D), indicating that CTSG activity contributes to leukemic cell invasion in established AML cell lines. A similar inhibitory effect was observed in multiple independent primary AML patient–derived cells, in which CTSG inhibition markedly suppressed cell invasiveness (Figure 5E). We assessed LTF protein expression in AML cells from four independent patients by Western blot (Figure 5F) and found that patient #4 exhibited markedly low LTF expression compared to the other samples. Consistently, this patient also showed reduced *Ctsg* expression (Figure 5G) and a diminished response to CTSGi treatment in the invasion assay (Figure 5E). These findings provide supportive evidence in primary AML samples that LTF may regulate leukemic cell invasion through downstream NSPs, further strengthening the clinical relevance of the LTF–NSPs axis.

**FIGURE 6:**
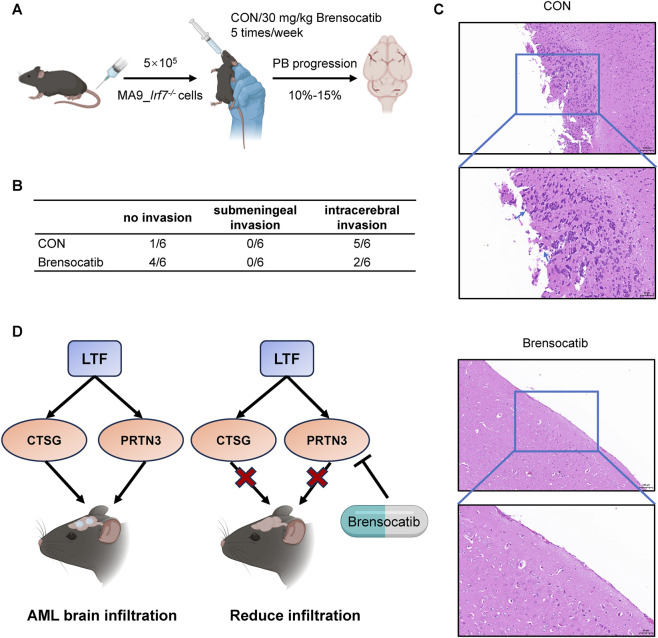
Pharmacological inhibition of NSPs suppresses AML CNS infiltration *in vivo*. **(A)** Schematic illustration of *in vivo* experimental design. 5 × 10^5^ MA9_Irf7^−/−^ leukemia cells were transplanted via tail vein injection into recipient mice. Mice were treated with vehicle control (CON) or Cathepsin C (CTSC) inhibitor brensocatib (30 mg/kg/day, five times per week) starting from day 14 post-transplantation until PB leukemia burden reached 10%–15%, at which point brains were collected for analysis. **(B)** Quantification of CNS leukemic infiltration in vehicle control and brensocatib-treated mice. CNS involvement was categorized as no invasion, submeningeal invasion, or intracerebral invasion based on histopathological evaluation of brain sections. Data are presented as the number of mice in each category (n = 6). **(C)** Representative HE staining of brain sections from control and brensocatib-treated mice. Low-magnification images are shown to provide anatomical context, with boxed regions enlarged in the corresponding panels. Infiltrating AML cells are indicated by blue arrows. **(D)** Schematic model illustrating the role of the LTF–NSPs axis in AML CNS infiltration. Left: LTF upregulates neutrophil serine proteases (CTSG and PRTN3) in leukemia cells, thereby promoting their CNS infiltration. Right: Pharmacological inhibition of NSPs activation using the CTSC inhibitor brensocatib reduces leukemic cell infiltration into the central nervous system.

Because Cathepsin C (CTSC) activates NSPs, including CTSG and PRTN3, we further evaluated the effect of NSPs inhibition *in vivo*. MA9_WT leukemia mice were administered Brensocatib orally (30 mg/kg, five times weekly) (Basso et al., 2023). Mice were sacrificed at matched time points when peripheral blood (PB) leukemia burden was comparable between vehicle-treated and brensocatib-treated groups. After 3 weeks, brain infiltration was substantially reduced (Figure 6A), with only 2/6 mice exhibiting intracerebral involvement compared to 5/6 in vehicle-treated controls (Figures 6B,C).

Collectively, these data demonstrate that LTF promotes AML cell CNS invasion via NSPs such as CTSG and PRTN3 (Figure 6D, left), and that inhibiting NSP activation effectively mitigates this process, providing a potential therapeutic strategy for CNS leukemia (Figure 6D, right).

## 4 Discussion

CNS leukemia, a form of extramedullary infiltration in acute leukemia, remains poorly understood and lacks effective targeted therapies. In this study, we identified *LTF* as a critical mediator of CNS infiltration in AML and elucidated its downstream regulation through neutrophil serine proteases (NSPs).

Using a multi-model machine learning approach, we constructed a prognostic framework for AML based on cytokine-regulated gene signatures. This model stratifies patients into high- and low-risk groups with distinct prognoses and captures key biological hallmarks of aggressive AML, including activated inflammatory pathways and altered immune microenvironments. Among the high-weight genes contributing to prognostic prediction, *LTF* emerged as a previously underappreciated factor associated with poor AML outcomes. Although the AML cohort used to construct the prognostic model did not contain information on CNS involvement, previous reports have shown that LTF is aberrantly expressed in hematologic malignancies (Dawson et al., 2024), particularly subtypes prone to CNS involvement (Oberg et al., 1987). Therefore, we inferred that LTF may serve as a potential risk factor for CNS leukemia, thereby justifying its functional evaluation in AML models with CNS tropism.

Reducing *LTF* expression in AML cells via RNA interference did not significantly affect proliferation but impaired clonogenic potential. More importantly, *LTF* knockdown markedly reduced leukemia cell invasiveness, both *in vitro* and *in vivo*, as evidenced by fewer CNS infiltration foci in mouse brains. Conversely, overexpression of *LTF* in AML cells with low baseline CNS infiltration enhanced their ability to invade brain tissue. While these studies were performed in AML models, they align with previously reported high *LTF* expression in CNS-invasive ALL, warranting further investigation in ALL models.

NSPs, including CTSG and PRTN3, are widely expressed in myeloid and leukemia cells. Their expression correlates with poor prognosis in AML (Alatrash et al., 2016) and promotes tissue infiltration through extracellular matrix degradation (Tan et al., 2013; Felix and Gaida, 2016). Our data demonstrate that NSPs act as downstream effectors of LTF, mediating leukemia cell invasiveness. The findings from multiple primary AML patient samples further support the clinical relevance of the LTF–NSPs axis in leukemia cell invasion. Together with the results observed in established cell lines, pharmacological inhibition of NSPs consistently suppressed the invasive capacity of primary AML cells derived from multiple independent patients. These data suggest that targeting NSP activity may represent a feasible strategy to limit leukemic cell dissemination.

Moreover, analysis of protein expression in primary samples revealed inter-patient heterogeneity in LTF levels. Notably, one patient exhibiting markedly low LTF expression also displayed reduced *Ctsg* expression and a diminished response to CTSG inhibition. Although limited by the small sample size, this observation is consistent with a potential functional association between LTF expression and NSPs activity in AML. Together, these findings provide preliminary evidence that the LTF–NSPs axis may contribute to the regulation of leukemic cell invasion in a patient-dependent manner, thereby reinforcing its potential clinical and translational significance.

Pharmacologic inhibition of NSPs, using Brensocatib, effectively reduced CNS infiltration in AML mice, highlighting their potential as therapeutic targets. Notably, NSPs are also aberrantly expressed in ALL and predict poor outcomes (Khan et al., 2018), suggesting that targeting NSPs could benefit a broader spectrum of hematologic malignancies with CNS involvement.

Currently, cytarabine and methotrexate remain the mainstay of CNS leukemia therapy (Wu et al., 2022). These drugs primarily eliminate leukemia cells that have already infiltrated the CNS, rather than preventing CNS invasion. Our findings suggest that even compounds with limited blood-brain barrier penetration, such as Brensocatib (Agency, 2025), can mitigate CNS invasion by inhibiting NSP activity in leukemia cells.

In summary, our study reveals that LTF promotes CNS infiltration of leukemia cells via serine proteases CTSG and PRTN3. Pharmacologic inhibition of NSPs effectively attenuates LTF-driven CNS invasion, uncovering a mechanistic axis linking LTF to downstream effectors. These results provide a rationale for targeting the LTF–NSP pathway as a potential strategy to prevent or treat CNS leukemia and highlight novel therapeutic avenues for high-risk AML and other CNS-invasive hematologic malignancies.

## Data Availability

The datasets presented in this study can be found in GitHub (https://github.com/HuasongYu/MachineLearn).
