# Supplementary material for: LTF promotes central nervous system leukemia progression via neutrophil serine proteases
Source: Front Pharmacol. 2026 Jul 1;17:1813396. doi: 10.3389/fphar.2026.1813396 (PMC13368923; doi:10.3389/fphar.2026.1813396)
Supplement: Supplementary file 1 [file Supplementaryfile1.docx]

**Supplementary Figure**

**

**

**Supplementary Fig.1 The Effect of the Number of Trees on the Error Rate in the RSF model.**

**Supplementary Tables**

**Supplementary Table 1. Reagents or Resources**

| **Reagents or Resources** | **Source** | **Identifier** |
| --- | --- | --- |
| **Cell line and Cell Culture** | | |
| HEK293T | ATCC | Cat#CRL-3216 |
| HL60 | ATCC | Cat#CCL-240 |
| THP1 | ATCC | Cat#TIB-202 |
| Iscove’s Modified Dulbecco’s Medium (IMDM) | Gibco | Cat#C12440500BT |
| Dulbecco's Modified Eagle Medium (DMEM) | Gibco | Cat#11995065 |
| Roswell Park Memorial Institute 1640 (RPMI-1640) Medium | Gibco | Cat#11875093 |
| Fetal bovine serum (FBS) | Gibco | Cat#16000044 |
| Penicillin–streptomycin | Solarbio | Cat#P1400-100 |
| Lymphocyte Separation Medium | Tbd Science | Cat#LTS1077 |
| Serum-free Freezing Medium | FUJIFILM | Cat#302-14686 |
| **Mice** | | |
| C57BL/6J | Beijing HFK Bioscience Co., Ltd. | |
| **Recombinant cytokines and Chemicals** | | |
| Recombinant Murine IL-6 (rmIL-6) | PeproTech | Cat#216-16 |
| Recombinant Murine IL-3 (rmIL-3) | PeproTech | Cat#213-13 |
| Recombinant Murine SCF (rmSCF) | PeproTech | Cat#250-03 |
| Recombinant Human SCF (rhSCF) | PeproTech | Cat#300-07 |
| recombinant Human Flt3 ligand (rhFlt3L) | PeproTech | Cat#300-19 |
| Recombinant Human Thrombopoietin (rhTPO) | PeproTech | Cat#AF-300-18-10 |
| Hieff Trans^®^ Liposomal 2000 | Yeason | Cat#40802ES03 |
| PEG-6000 | Solarbio | Cat#P8250 |
| polybrene | Beyotime | Cat#C0351 |
| Brensocatib | Selleck | Cat#E1188 |
| RBC lysis buffer | Solarbio | Cat#R1010 |
| TRIzol reagent | Invitrogen | Cat#15596018CN |
| RIPA buffer | Solarbio | Cat#R0020 |
| protease inhibitors | Yeason | Cat#20124ES03 |
| 5× loading buffer | ABclonal | Cat#RM00001 |
| Bovine Serum Albumin | Solarbio | Cat#A8020 |
| **Critical Commercial Assays** | | |
| CD117 microbeads | Miltenyi Biotec | Cat#130-091-224 |
| MethoCult M3434 methylcellulose | StemCell Technologies | Cat#03434 |
| BeyoGold™ Transwell inserts | Beyotime | Cat#FTW067-12Ins |
| Matrix-Gel™ | Beyotime | Cat#C0372 |
| ABScript III RT Master Mix with gDNA Remover | ABclonal | Cat#RK20429 |
| 2× Universal SYBR Green Fast qPCR Mix | ABclonal | Cat#RK21203 |
| BCA Protein Assay Kit | Thermo Scientific | Cat#23227 |
| 4%–20% SurePAGE gels | GenScript | Cat#M00657 |
| ultra-sensitive ECL substrate | CotyBioTech | Cat#KE0126 |
| **Antibodies** |  |  |
| β-actin | Sigma-Aldrich | Cat#A5316 |
| LTF | Invitrogen | Cat#PA5-95513 |
| Goat Anti-Mouse IgG H&L (HRP) | Abcam | Cat#ab205719 |
| Goat Anti-Rabbit IgG (H+L), HRP | Invitrogen | Cat#31460 |
| **Software and Platforms** | | |
| GraphPad Prism 9 | https://www.graphpad.com/ | |
| FlowJo_V10 | https://www.flowjo.com/ | |
| The Cancer Genome Atlas (TCGA) | https://portal.gdc.cancer.gov/ | |
| NCBI Gene Expression Omnibus (GEO) | https://www.ncbi.nlm.nih.gov/geo/ | |

**Supplementary Table 2. Primers for qRT-PCR**

| **Primers** | **Forward (5’-3’)** | **Reverse (5’-3’)** |
| --- | --- | --- |
| Ltf | CCAGGCCATTGTGACAAACAG | CACGACTGCTACCGCATAGT |
| Ctsg | TCTGCCTTCAGGGGTGATTC | TCCATAGGAGACGATGCCCT |
| Prtn3 | CGTGCTTCTCCTCCAGCTAAA | CACCGTGACGTTCAGTTCCT |
| Gapdh | TTGGCCGTATTGGGCGCCTG | CACCCTTCAAGTGGGCCCCG |

**Supplementary Table 3. Primers for shRNA**

| **shRNA** | **shRNA sequences** |
| --- | --- |
| shNC | CCTAAGGTTAAGTCGCCCTCG |
| shLtf-1 | GAGGAGTTACCAAATAAA |
| shLtf-2 | TCCATACAGAACCTGAATAAA |
| shLtf-3 | GACCAGATCCTGCAAATTTA |

**Supplementary Table 4. Characteristics of the AML patients used in the study**

| **AML patients** | **Gender** | **Age** | **Tissue** | **Disease Status** | **CNS Involvement** |
| --- | --- | --- | --- | --- | --- |
| #1 | Female | 29 | Bone marrow | Relapsed/Refractory | N/A |
| #2 | Male | 37 | Bone marrow | Relapsed/Refractory | N/A |
| #3 | Female | 72 | Bone marrow | Relapsed/Refractory | N/A |
| #4 | Female | 38 | Bone marrow | Relapsed/Refractory | N/A |
