# Supplementary figures and images for: LTF promotes central nervous system leukemia progression via neutrophil serine proteases
Source: Front Pharmacol. 2026 Jul 1;17:1813396. doi: 10.3389/fphar.2026.1813396 (PMC13368923; doi:10.3389/fphar.2026.1813396)

Full Western blot images

Refer to Figure 2C

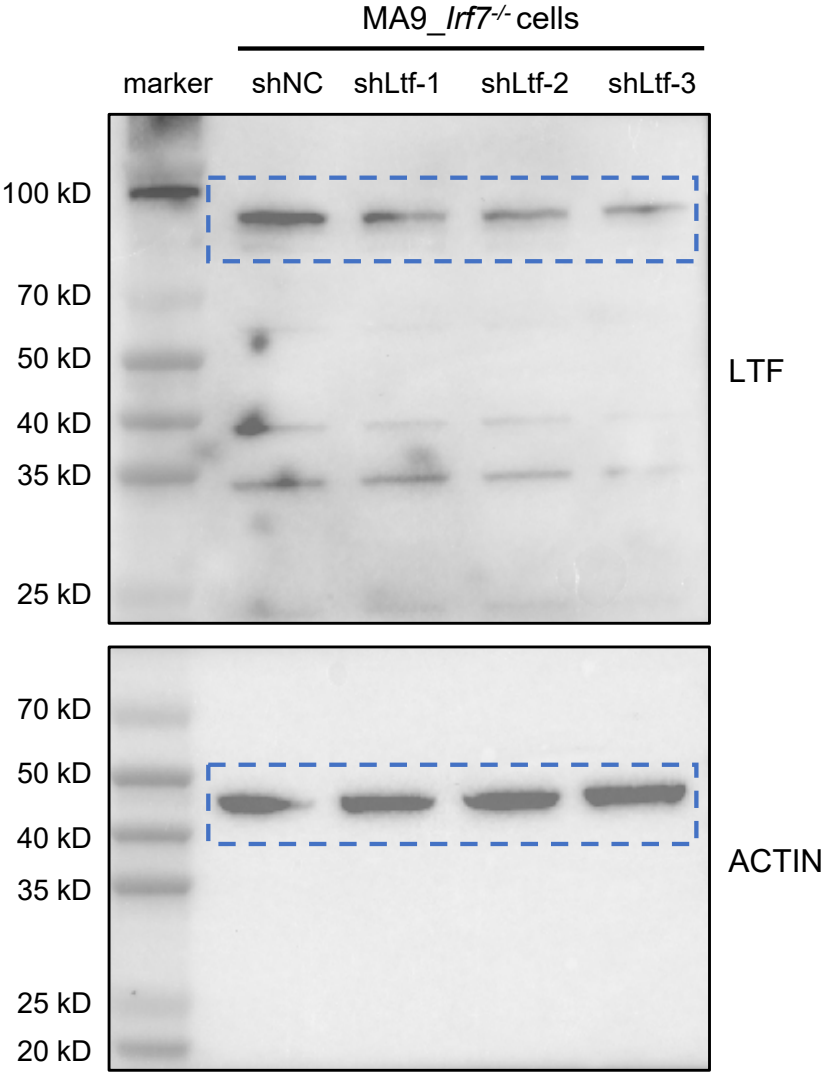

Refer to Figure 4C

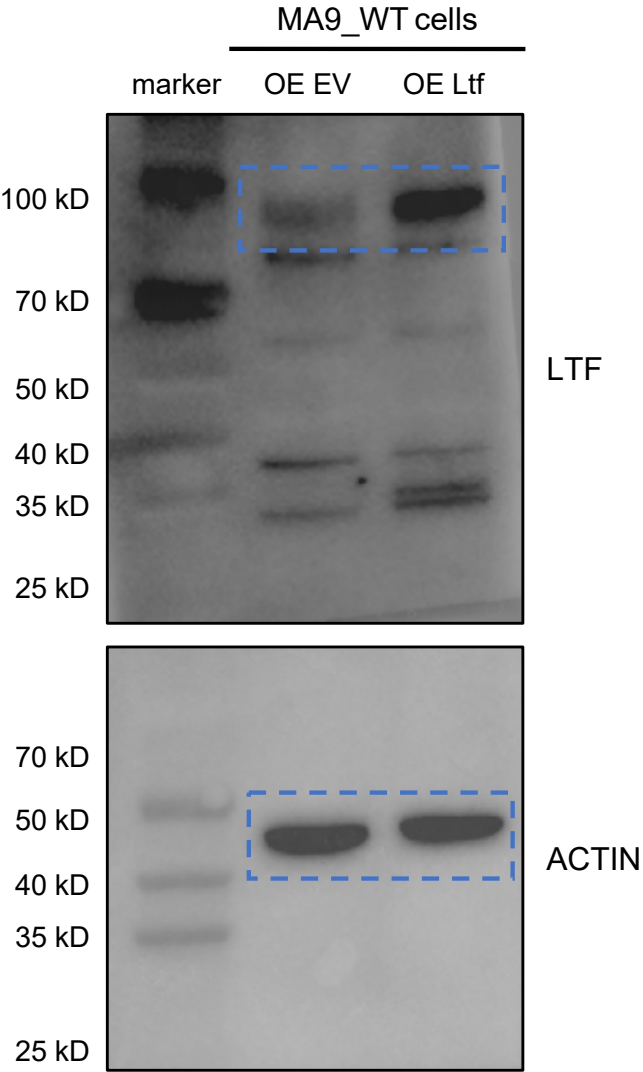

Refer to Figure 5F

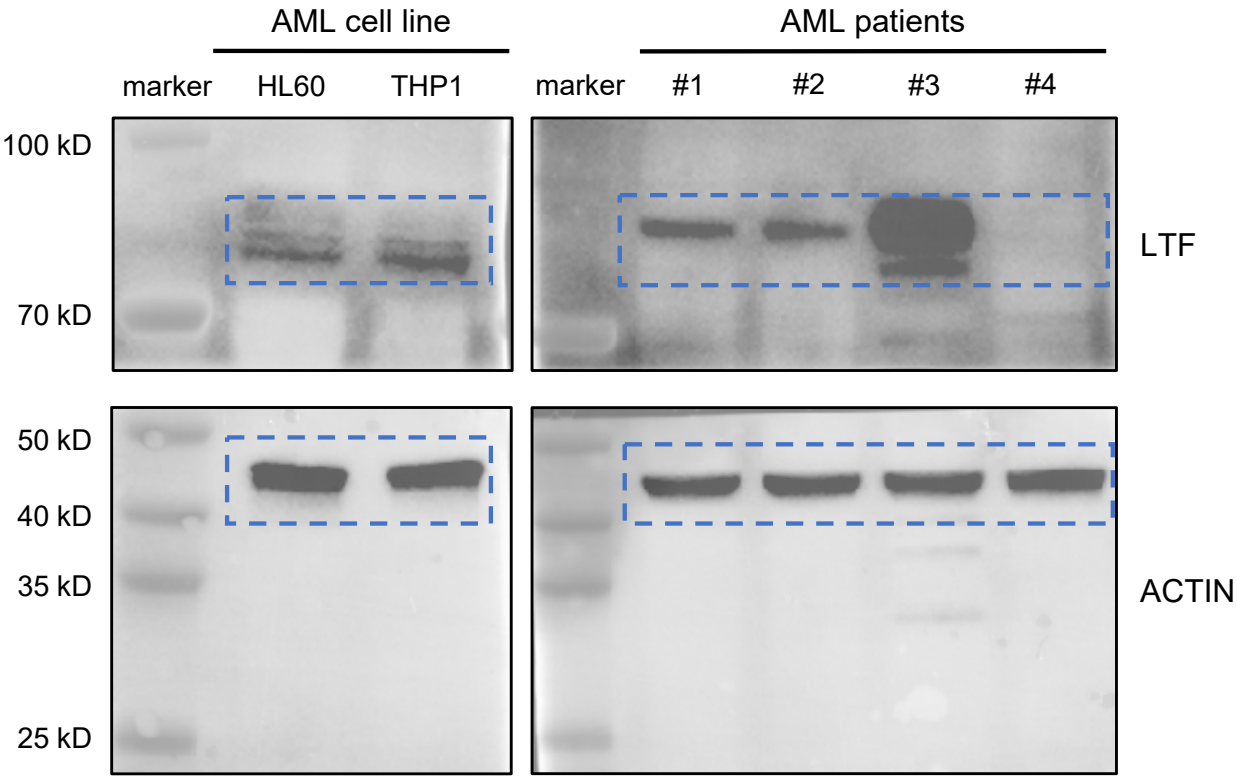

Supplement: Supplementary file 2 [file Image2.PDF]
