# Supplementary material for: LTF promotes central nervous system leukemia progression via neutrophil serine proteases
Source: Front Pharmacol. 2026 Jul 1;17:1813396. doi: 10.3389/fphar.2026.1813396 (PMC13368923; doi:10.3389/fphar.2026.1813396)

All animals HE overview images

Refer to Figure 3B

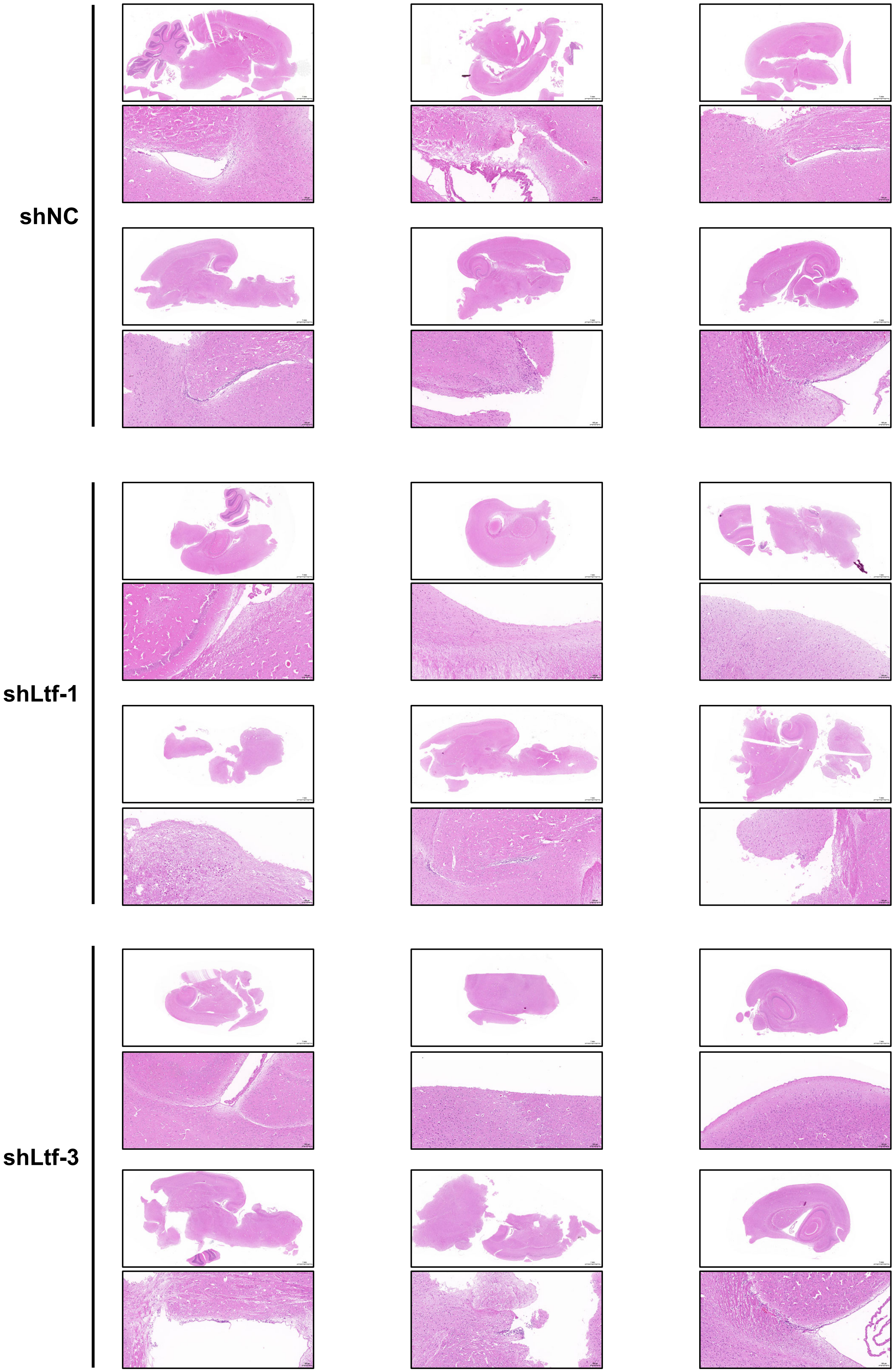

All animals HE overview images

Refer to Figure 4E

OE EV

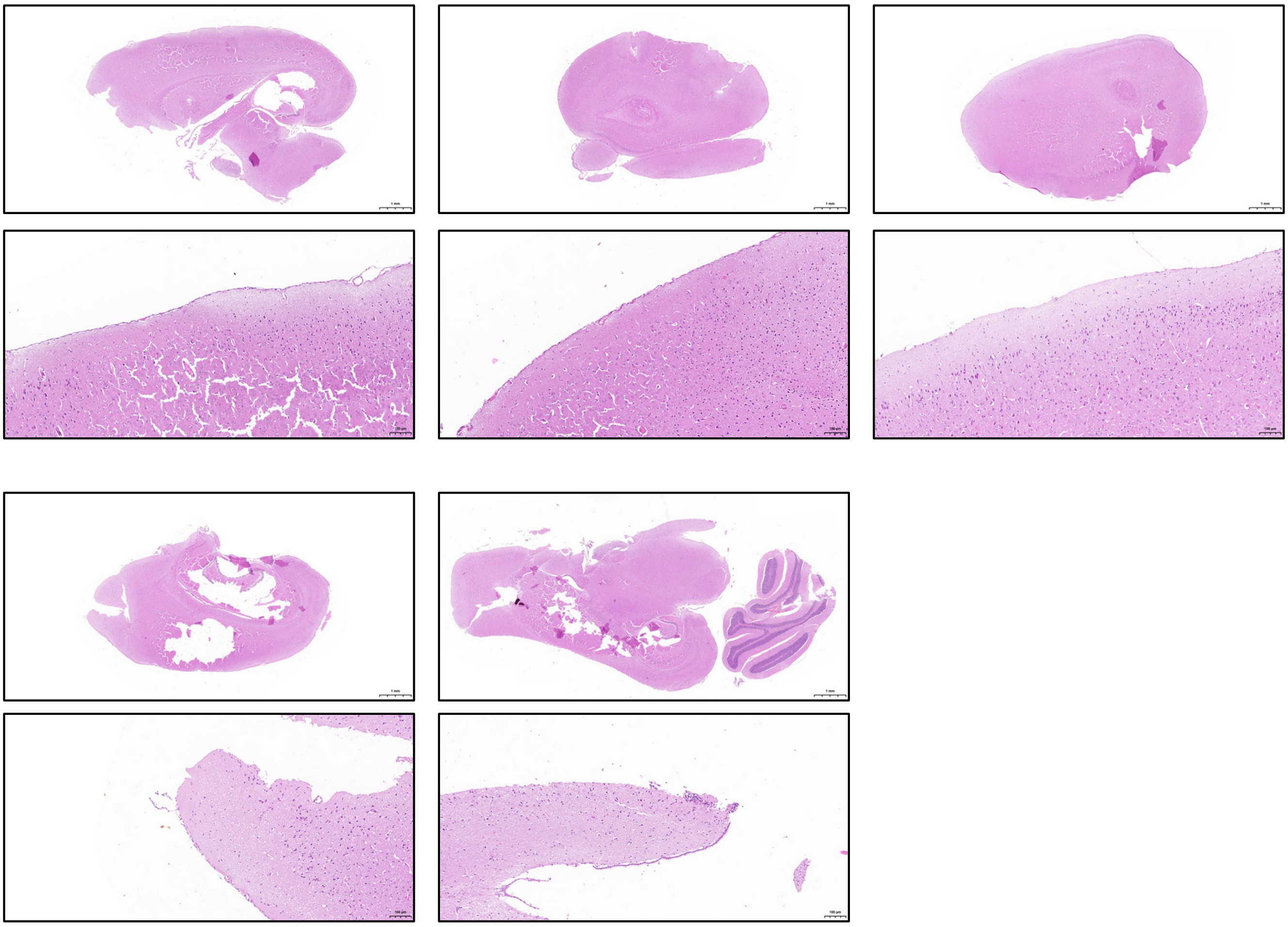

OE Ltf

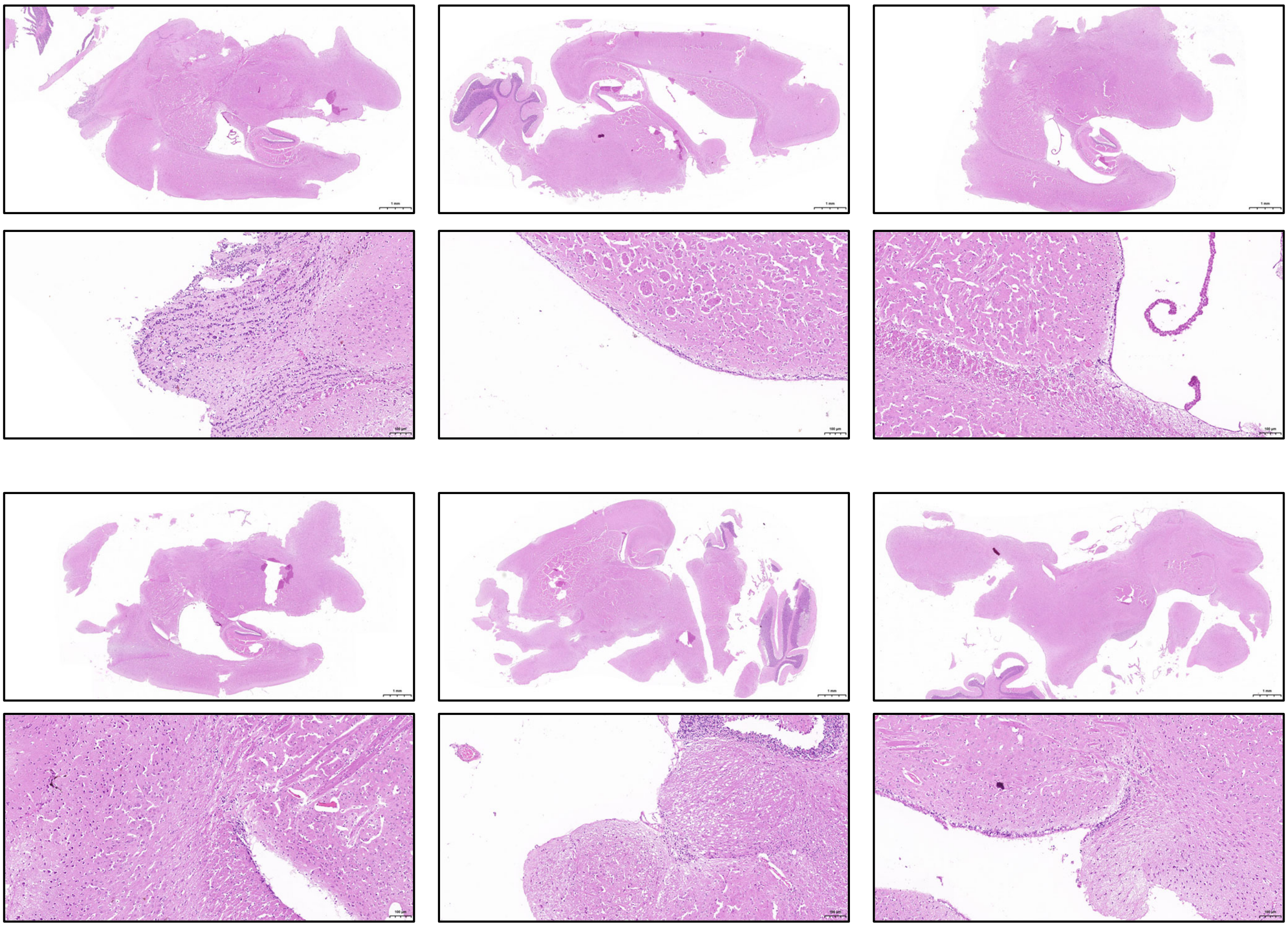

All animals HE overview images

Refer to Figure 6C

CON

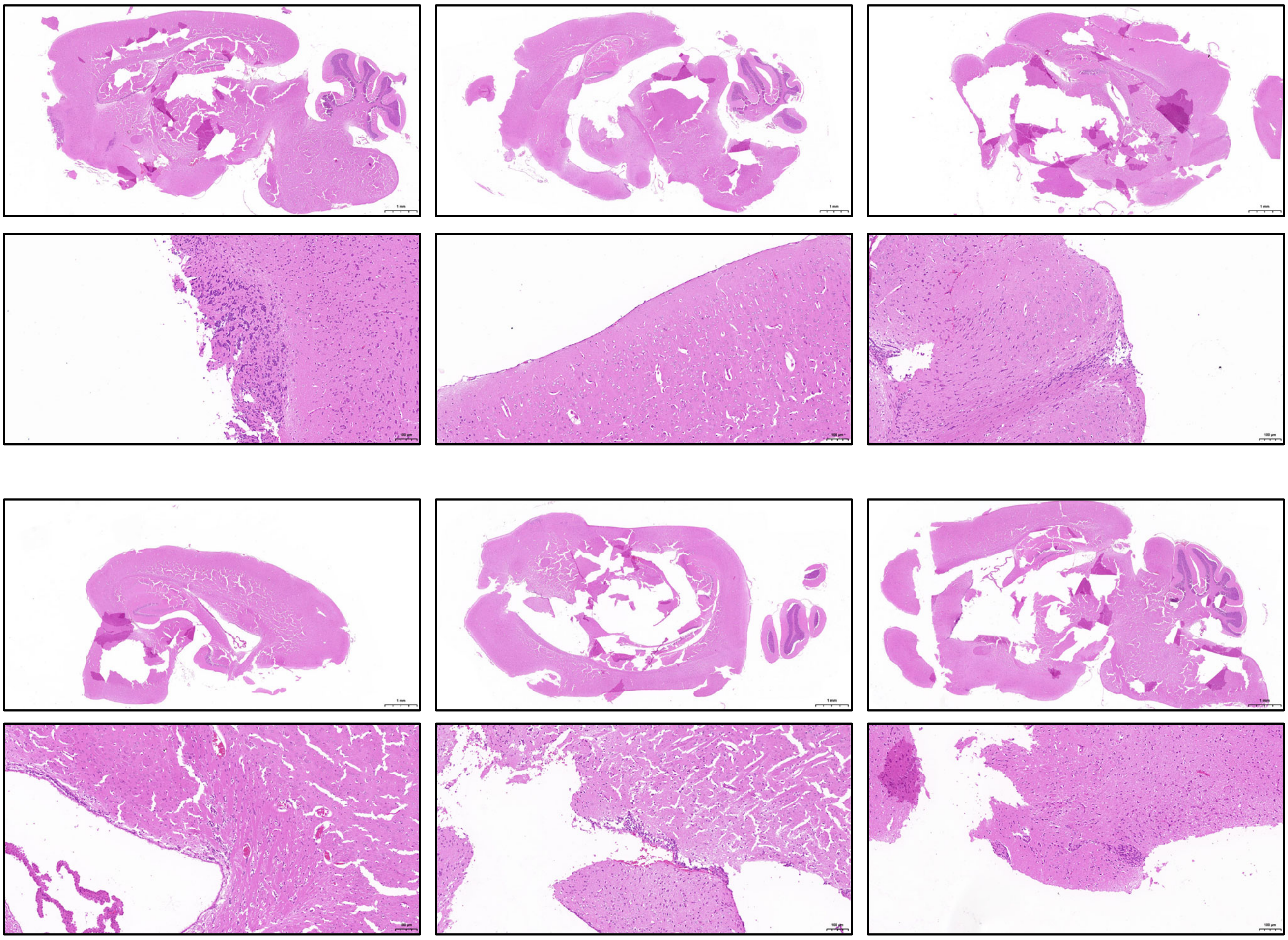

Brensocatib

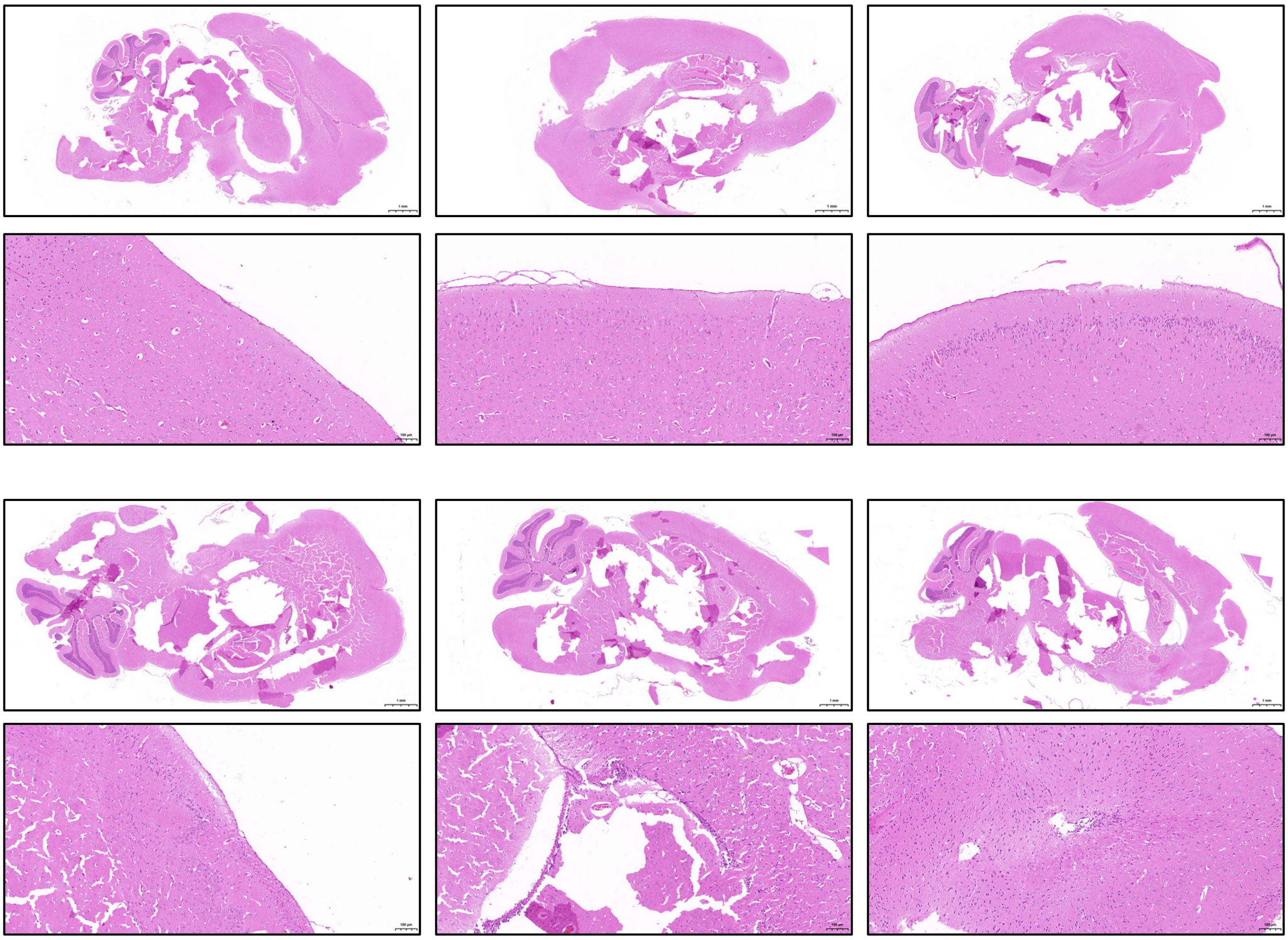

Supplement: Supplementary file 3 [file Image1.PDF]
